# Benign gallbladder disease is a risk factor for colorectal cancer, but cholecystectomy is not: A propensity score matching analysis

**DOI:** 10.3389/fonc.2022.1008394

**Published:** 2022-12-08

**Authors:** Qiong Qin, Wei Li, Ao Ren, Rong Luo, Shiqiao Luo

**Affiliations:** ^1^ Department of Hepatobiliary Surgery, The First Affiliated Hospital of Chongqing Medical University, Chongqing, China; ^2^ Medical Examination Center, The First Affiliated Hospital of Chongqing Medical University, Chongqing, China

**Keywords:** benign gallbladder disease, cholecystectomy, colorectal cancer, risk factor, propensity score matching analysis

## Abstract

**Background:**

Previous studies reported controversial results on the relationship between cholecystectomy (CHE) and colorectal cancer (CRC). We hypothesized that gallbladder disease (GBD), instead of cholecystectomy, increased the risk of CRC. We aimed to investigate the incidence of benign gallbladder disease (BGBD) and CHE in CRC patients and local adults undergoing annual health examination by analyzing large data from a tertiary hospital in southwest China.

**Methods:**

A propensity score matching (PSM) analyzed, retrospective study from January 1, 2013, to August 31, 2020, including 7,471 pathologically confirmed CRC patients and 860,160 local annual health examination adults in the First Affiliated Hospital of Chongqing Medical University, was conducted. The prevalence of BGBD and the CHE rate were analyzed before and after a 1:1 PSM.

**Results:**

Of the 7,471 CRC patients, 7,160 were eligible for the case group. In addition, 860,160 local health examination adults were included for comparison. The incidence of BGBD was higher in the CRC patients than in the local adults (19.2% vs. 11.3%, *P* < 0.001), but no significant difference in CHE rate existed between the case group and the control group (5.0% vs. 4.8%, *P* = 0.340). In the subgroup analysis, patients with BGBD had a higher risk of colon cancer than rectal cancer (20.4% vs. 18.2%, *P* = 0.024) and more significantly in the right colon (*P* = 0.037). A weakly positive correlation between CHE and right colon cancer was observed before PSM but no longer existed after PSM (*P* = 0.168).

**Conclusions:**

Benign gallbladder disease was positively correlated with colorectal cancer, especially right colon cancer. Cholecystectomy did not increase the risk of colorectal cancer.

## Introduction

Colorectal cancer (CRC) ranks third among lethal cancers worldwide, accounting for nearly 10% of cancer-related deaths each year. It is the second most common cancer in women and the third most common cancer in men (1–4). In China, both the morbidity and mortality of CRC are fifth among cancers (5). Anatomically, based on tumor location, CRC can be divided into right colon cancer (also called proximal colon cancer, including the ascending colon and the front two-thirds of the transverse colon), left colon cancer (also called distal colon cancer, including the posterior third of the transverse colon, descending colon and sigmoid colon) and rectal cancer (4, 6). In general, distal colon cancer is more common than proximal colon cancer, and patients with distal colon cancer are younger than those with proximal colon cancer. In terms of sex distribution, men are more likely to develop CRC than women, possibly due to sex hormone effects. The disparity is more pronounced in older patients. However, there are more women diagnosed in the right colon than men, and the reverse is true in the left colon (6, 7). In 2017, the incidence of CRC was 46.9/100,000 in men and 35.6/100,000 in women in the United States, nearly double that in China (28.64/100,000 in men and 19.33/100,000 in women) (8). CRC patients are getting younger at diagnosis, with the median age of diagnosis dropping from 72years during 1988 and 1989 to 66years during 2015 and 2016. From 2012 to 2016, the prevalence increased by 9% to 10% annually among people 50years old or older, and by up to 24% annually among people under 50years old. From 2008 through 2017, the mortality rate for CRC patients over 65years declined by 35% per year, for those aged 50 years to 64years by 0.6% per year but increased by 1.3% per year for those aged under 50years (6, 9). With the incidence and mortality of CRC increasing annually and patients getting younger, it is crucial to identify the risk factors for CRC prevention and treatment.

Benign gallbladder disease (BGBD) is the most common cause of nonmalignant gastrointestinal death and can severely affect the quality of life (10–13). Clinically, common benign gallbladder diseases include gallstones, cholecystitis, and gallbladder polyps, of which gallstones are referred to as calculous diseases (CD), and cholecystitis and gallbladder polyps are collectively referred to as acalculous disease (ACD) (14). BGBD affects 10%~20% of the global population, 10%~30% in Western countries and 5.9%~21.9% in Asian countries. The prevalence of BGBD differs from 4.2% to 13.11% in different regions of China and varies from 10.45% to 11.64% in the Han population (10, 11, 15–17). In the general population, BGBD prevalence is higher in females than in males (10, 13, 14) and higher in older people than in younger people. The prevalence increases with age (14, 18). Studies by Shaffer (19) and Liu et al. (20) revealed that the incidence rate was 4–10 times and 3.02–3.11 times higher in those over 40 and over 50 than in those under 40 and under 50, respectively. Currently, cholecystectomy (CHE) is the standard treatment for symptomatic BGBD and BGBD with complications, especially gallstones and large gallbladder polyps (21). Because of high incidence of BGBD, cholecystectomy is one of the most performed procedures in surgery. There are approximately 300,000 cholecystectomies performed annually in the United States (22). Although lack of available data, more cholecystectomies may be performed in China, considering similar incidence and more population.

Studies on the relationship between BGBD or CHE and CRC can be traced back to 1978 (23, 24). Some of the current studies suggested a positive correlation with digestive system cancer (25–28), and some believed no correlation existed (29–31). Some studies have shown that the association varies by different tumor sites (32–34). Researchers who proposed a positive correlation believed in the following mechanisms. First, the two diseases shared the same risk factors (11, 33, 35). Risk factors for BGBD, including old age (15, 18), obesity (12), hypercholesterolemia (36, 37), smoking (35), diabetes (13, 38), low-fiber and high-fat diet, and low physical activity (39, 40), are also well-known risk factors for large bowel cancer. Ernst J. Kuipes et al. (4) revealed a 1 unit increased of body mass index (BMI) and a 2–3% increase in CRC risk. Sencond, alterations in bile flow, long-term inflammatory stimulation, and complications caused by BGBD can promote the occurrence of CRC (41–43). Third, Hill, MJ et al. (44) suggested that the gallbladder lost its storage function after CHE and increased secondary bile acids (SBAs), which continued to be secreted into the intestine without food dilution, induced carcinogenesis (45). Elevated levels of bile acids and derivatives have been found in stool from CRC patients and patients who accepted CHE (44). Some studies found that the correlation varied depending on sex and tumor site. A positive relationship in women has been confirmed by many studies, especially in the proximal colon. However, such a relationship has not been proven in men (33, 34). These views were also borne out in some Chinese studies (46). Although many studies suggested a positive correlation, studies that suggested no correlation were not uncommon (29, 31). Despite nearly half a century of exploration, the relationship remains a mystery. Given high frequency of BGBD and CHE worldwide, more evidence is needed to determine whether BGBD and CHE increase the risk of future CRC. A preliminary study in our data indicated there were much more CRC patients with BGBD than CRC patients with history of CHE. Based on a hypothesis that benign gallbladder disease, rather than cholecystectomy, can increase the risk of colorectal cancer, we carry out the analysis to provide more evidence to reveal relationship between benign gallbladder disease or loss of gall bladder and colorecatal cancer.

## Materials and methods

### Study population

**Figure 1:**
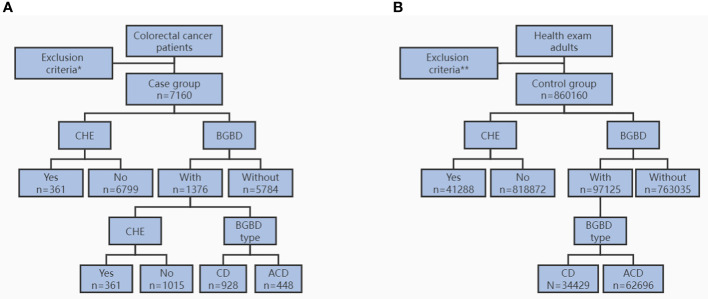
Flow diagram for study population of case group **(A)** and control group **(B)**. CHE, cholecystectomy. BGBD, benign gallbladder disease. CD, calculous disease. ACD, acalculous disease. * age < 18 years, BMI < 15.0kg/m^2^, multifocal CRC, other cancer, and incomplete data. ** age < 18 years, BMI < 15.0kg/m^2^, any cancer, and incomplete data.

In this large, single-center, retrospective study, a total of 7,471 CRC patients admitted to the First Affiliated Hospital of Chongqing Medical University between January 1, 2013, and August 31, 2020, were screened, and 7,160 of them were eligible for the case group. A total of 860,160 people who visited the Medical Examination Center in the same period were included in the control group (**Figure 1**). For the case group, clinicopathological data, including sex, age, body mass index (BMI), tumor location, and time of CRC diagnosis, were collected. By reviewing abdominal ultrasound, computerized tomography (CT), magnetic resonance imaging (MRI), and electronic medical records (EMR), CRC patients with a history of BGBD were identified, including symptomatic and asymptomatic patients. The time of BGBD diagnosis, BGBD type, CHE or not, and time of CHE were included. For the control group, BGBD and CHE information was included through retrieval in the dedicated electronic system of the Medical Examination Center. In this study, BGBD types included gallstones, cholecystitis, and gallbladder polyps. The former subtype was called CD, and the latter two subtypes were collectively referred to as ACD.

### Inclusion and exclusion criteria

The inclusion criteria for the case group included: (1) age ≥ 18 years; (2) BMI ≥ 15.0kg/m^2^; (3) histologically or cytologically confirmed CRC. The exclusion criteria for the case group included: (1) age < 18 years; (2) BMI < 15kg/m^2^; (3) multifocal CRC; (4) other cancer; and (6) incomplete data.

The study population in the control group included an asymptomatic health examination population and a symptomatic physical check-up population during the same period as the case group. The inclusion criteria of the control group were as follows: (1) age ≥18 years and (2) BMI ≥15.0kg/m^2^. Exclusion criteria of the control group included (1) age < 18 years; (2) BMI < 15kg/m^2^; (3) any cancer; and (4) incomplete data.

### Ethical approval

This study was approved by the Clinical Research Ethics Review Committee of The First Affiliated Hospital of Chongqing Medical University (registration number: 2021-770).

### Propensity score matching analysis

To decrease the effect of selection bias and confounding factors and increase comparability between subgroups, we performed propensity score matching (PSM) analysis. PSM is a statistical method that can be used to balance interference factors between groups in observational studies, following the law of counterfactual reasoning (47). The PSM consists of the following steps: (1) Use the logistic regression model to calculate propensity scores. (2) Score matching is performed by nearest neighbor matching (NNM), radius matching, or kernel matching. (3) Evaluate the balance after matching. (4) Calculate the average intervention effect (ATT). (5) Conduct sensitivity analysis (48). All steps can be implemented in SPSS software. In our study, we calculated propensity scores by applying the sex, age, and BMI of patients in the case group to a logistic regression model and evaluated the goodness of fit with the caliper value level of 0.002. Finally, one-to-one PSM was achieved (without replacement). Then, we analyzed subgroups before and after 1:1 PSM. The process of PSM was implemented in Microsoft Office 2019 and SPSS^®^ version 23.0 (IBM, Armonk, New York, USA).

### Statistical analysis

All statistical analyses were performed in SPSS^®^ version 23.0 (IBM, Armonk, New York, USA). The normality of continuous variables was tested using the P-P graph, histogram, and single-sample Kolmogorov−Smirnov test. PSM was used to match the patient’s sex, age, and BMI. Continuous variables are presented as the mean values with ranges, and categorical variables are presented as frequencies with percentages. *P <* 0.05 was used to denote a statistically significant difference.

The chi-square test was used to compare all categorical variables. Before PSM, an independent sample t test or one-way analysis of variance was employed to compare normally distributed data, and the Mann−Whitney U test was used to compare nonnormally distributed data. After PSM, the paired sample t test was used for normally distributed variables, and the Wilcoxon test or the Friedman test was used for nonnormally distributed data.

## Results

### Case and control group analysis

Of the 7,160 CRC patients in the case group, 1,376 (19.2%) had a history of BGBD, and 5,784 (80.8%) did not. Among them, 361 (5.0%) patients had previously undergone cholecystectomy, and 6,799 (95.0%) patients had not. A total of 860,160 local annual health examination adults were enrolled in the control group, of which 97,125 (11.3%) had BGBD and 763,035 (88.7%) did not. Among them, 41,288 (4.8%) accepted cholecystectomy, and 818,872 (95.2%) did not. The prevalence of BGBD in the case group was significantly higher than that in the control group (19.2% vs. 11.3%, *P* < 0.001, **Table 1**). However, there was no significant difference in the CHE rate between the case and the control groups (5.0% vs. 4.8%, *P* = 0.340, **Table 1**).

**Table 1:** Incidence of BGBD and CHE between colorectal cancer patients and health examination adults.

	Case group (n = 7160)	Control group (n = 860160)	P value
BGBD^*^, n (%)			<0.001
with	1376 (19.2)	97125 (11.3)	
without	5784 (80.8)	763035 (88.7)	
CHE^*^, n (%)			0.340
Yes	361 (5.0)	41288 (4.8)	
No	6799 (95.0)	818872 (95.2)	

^*^BGBD, benign gallbladder disease. CHE, cholecystectomy.

### Baseline characteristics of subgroups in the case group

The characteristics of the subgroups based on tumor location showed that the features of right colon cancer were significantly different from those of left colon cancer and rectal cancer. Obvious differences were found in sex, age, and BMI distribution among the three subgroups (*P* < 0.001, **Table 2**). Generally, CRC was more common in men than women, but right colon cancer was more common in women than left colon cancer and rectal cancer (*P* < 0.001). On average, patients with rectal cancer and left colon cancer were younger than those with right colon cancer (62.6years vs. 63.6years vs. 64.5years, respectively, *P* < 0.001, **Table 2**). The right colon cancer had a lower BMI than the left colon cancer and rectal cancer (BMI were 21.9kg/m^2^ vs. 22.6kg/m^2^ vs. 22.5kg/m^2^, respectively, *P* < 0.001, **Table 2**), which was consistent with the clinical phenomenon—right colon cancer with mainly systemic symptoms and left colon cancer with mainly intestinal obstruction symptoms. As shown in **Table 2**, patients with previous BGBD were more likely to develop right colon cancer (*P* = 0.004), regardless of BGBD subtype (*P* = 0.074). Notably, no difference in CHE rate among the different locations of CRC existed (*P* = 0.074). However, the sex, age, and BMI of the three subgroups were not balanced at baseline, which made the above conclusions inconclusive and required further analysis by PSM.

**Table 2:** Baseline characteristics of different tumor locations in colorectal cancer.

	Right colon = 1473	Left colon = 1716	Rectum = 3971	P value
Sex, n (%)				<0.001
Male	780 (53.0)	1089 (63.5)	2517 (63.4)	
Female	693 (47.0)	627 (36.5)	1454 (36.6)	
Age, median (range), years	64.5 (18-93)	63.6 (21-96)	62.6 (20-96)	<0.001
BMI, median (range), kg/m^2^	21.9 (15.0-34.9)	22.6 (15.0-36.1)	22.5 (15.0-41.6)	<0.001
BGBD^*^, n (%)				0.004
With	318 (21.6)	347 (20.2)	711 (17.9)	
Without	1155 (78.4)	1369 (79.8)	3260 (82.1)	
CHE^*^, n (%)				0.074
Yes	91 (6.2)	85 (5.0)	185 (4.7)	
No	1382 (93.8)	1631 (95.0)	3786 (95.3)	
BGBD^*^ type, n (%)	318 (100)	347 (100)	711 (100)	0.074
CD^*^	231 (72.6)	231 (66.6)	466 (65.5)	
ACD^*^	87 (27.4)	116 (33.4)	245 (34.5)	

^*^BGBD, benign gallbladder disease. CHE, cholecystectomy. CD, calculous disease. ACD, acalculous disease.

### Subgroup analysis before and after PSM

Information was obtained from the comparison of colon cancer and rectal cancer (**Table 3**). Before PSM, an imbalance was found at baseline for sex, age, and BMI between the two groups. A higher proportion of BGBD was found in colon cancer patients than in rectal cancer patients (20.9% vs. 17.9%, *P* = 0.002), but no significant difference in CHE rate existed between the two groups (5.5% vs. 4.7%, *P* = 0.098). After PSM, factors including sex, age, and BMI, were reconciled. Statistically significant differences still existed in the prevalence of BGBD between colon cancer and rectal cancer (20.4% vs. 18.2%, *P* = 0.024). The CHE rate remained not significantly different (5.2% vs. 4.9%, *P* = 0.562).

**Table 3:** Analysis between colon cancer and rectal cancer before and after PSM.

	Before PSM			After PSM		
	Colon = 3189	Rectum = 3971	P value	Colon = 3106	Rectum = 3106	P value
Sex, n (%)			<0.001			0.394
Male	1869 (58.6)	2517 (63.4)		1859 (59.9)	1826 (58.8)	
Female	1320 (41.4)	1454 (36.6)		1247 (40.1)	1280 (41.2)	
Age, median (range), years	64.0 (18-96)	62.6 (20-96)	<0.001	63.6 (18-95)	63.4 (20-96)	0.497
BMI, median (range), kg/m^2^	22.3 (15.0-36.1)	22.5 (15.0-41.6)	0.016	22.2 (15.0-36.1)	22.3 (15.0-36.6)	0.461
BGBD^*^, n (%)			0.002			0.024
With	665 (20.9)	711 (17.9)		634 (20.4)	564 (18.2)	
Without	2524 (79.1)	3260 (82.1)		2472 (79.6)	2542 (81.8)	
CHE^*^, n (%)			0.098			0.562
Yes	176 (5.5)	185 (4.7)		162 (5.2)	152 (4.9)	
No	3013 (94.5)	3786 (95.3)		2944 (94.8)	2954 (95.1)	

^*^BGBD, benign gallbladder disease. CHE, cholecystectomy.

Further analysis was performed before and after PSM 1:1 matching among right colon cancer, left colon cancer, and rectal cancer. As shown in **Supplementary Tables 1**-**3**, the three groups of patients were unbalanced at baseline. There was no difference in BGBD prevalence or CHE rate between right and left colon cancer before and after PSM analysis (**Supplementary Table 1**). Compared with rectal cancer, the prevalence of BGBD and CHE was higher in right colon cancer, but only the difference in BGBD remained after matching, and the difference in CHE disappeared (*P* = 0.037 and 0.168, respectively, **Supplementary Table 2**). There was no difference in the incidence of BGBD and CHE between left colon cancer patients and rectal cancer patients after matching (*P* = 0.126 and 0.523, respectively, **Supplementary Table 3**).

## Discussion

We had investigated association between benign gallbladder disease, cholecystectomy and colorectal cancer in a large sample, PSM-matched, case-control study. Our study revealed that benign gallbladder disease was positively associated with colorectal cancer, and this correlation was more pronounced in right colon cancer, which remained consistent before and after PSM analysis. However, our study did not found an increased risk of colorectal cancer caused by cholecystectomy. Before PSM, cholecystectomy was slightly positively correlated with right colon cancer, but this correlation no longer existed after matching the sex, age and BMI. Thus, cholecystectomy itself was not associated with the development of colorectal cancer. This might be explained by the reason that carcinogenic factors were formed as early as the occurrence of BGBD.

Previous studies, mainly case-control studies, cohort studies and inventory surveys, explored the relationship between BGBD, CHE and CRC but the results were controversial. Some studies revealed that both BGBD and CHE were risk factors of CRC and more closely related to proximal colon cancer (30, 49, 50), some studies revealed a positive correlation between BGBD and CRC but no correlation between CHE and CRC (51, 52), while some studies revealed an opposite result (27, 53). There were also studies revealed that neither BGBD nor CHE was associated with CRC (54, 55). Interestingly, Chen et al. (56) revealed a negative association between CHE and CRC through a long-term follow-up cohort study and believed that CHE was a protective factor for CRC. Our results favor that the BGBD, not the CHE, is a risk factor of CRC.

Studies supporting positive correlation between BGBD or CHE and CRC had further explored possible carcinogenic mechanisms.One widely accepted mechanism was that BGBD and CRC shared common risk factors, such as old age (15, 18), obesity (12), hypercholesterolemia (36, 37), smoking (35), diabetes (13, 38), low-fiber and high-fat diet (57), and low physical activity (39). In addition, studies by Almond, HR (45) and Adler et al. (58) revealed that shrinkage of the bile acid pool, changed bile lipid composition, increased secretion of secondary bile acids (SBAs), and increased enterohepatic circulation of bile acids were observed in patients with benign gallbladder disease, which might account for the development of colorectal cancer. It has been confirmed that SBAs in the stool of CRC patients (44), BGBD patients (59) and post-cholecystectomy patients (60) are significantly higher than those in normal people. SBA has been proved with strong carcinogenicity, its carcinogenic activities mainly occur through the following mechanisms. SBA inhibited peripheral blood lymphocytes and colonic mucosa lamina propria lymphocytes, reducing the secretion of secretory immunoglobulin A (SIgA) to weaken intestinal immune function, and the damaged intestinal mucosal barrier had increased permeability and susceptibility to carcinogens (61). SBA interfered with the detoxification of glutathione S-transferase (GST) against exogenous carcinogens (62). SBA promoted carcinogenesis and increased the invasive effect of cancer cells on blood vessels by activating AP21 through the protein kinase C (PKC) signaling pathway (63). SBA activated phosphatidylinositol 3 kinase (PI3K) through the epidermal growth factor receptor (EGFR) signaling pathway, regulating cell proliferation and apoptosis, and inducing carcinogenesis (64). SBA also destroyed the DNA stability of intestinal epithelial cells through oxidation, mutagenesis and transformation activities (65), resulting in biological toxicity. Due to an increase of highly carcinogenic SBAs after BGBD diagonsed or CHE performed and high concentration of SBAs in the stool of CRC patients, scholars had to speculate that BGBD or CHE might promote the occurrence of CRC through secondary bile acids.

However, Simmons (66) and Shaffer et al. (67) found that the bile lipid composition tended to normalize after cholecystectomy. They thought that carcinogenic factors were formed as early as BGBD occurred and cholecystectomy was only a treatment strategy after BGBD diagnosed. Cholecystectomy could not correct the carcinogenic effects of BGBD but itself was not a risk factor for CRC. Researches of Almond (45) and Metzger et al. (58) also revealed that bile acid pools, kinetics and diurnal variation of bile lipid composition did not significantly change before and after cholecystectomy. Thus, we suspected the opinion that cholecystectomy was a risk factor of colorectal cancer. Conversely, based on the bile lipid composition normalizing tendency, we hypothesized that cholecystectomy was a “correction” measure and protective factor for colorectal cancer. Finally, our study successfully provided convincing evidence that cholecystectomy was not a risk factor of colorectal cancer.

There were some obvious strengths of our study. First, this study had a large sample size in both the colorectal cancer group and the local annual health examination group, and the total number of participants was far larger than that in most previous studies. Second, we selected the population undergoing annual health examination as the control group, which could be representative of the local adult population. Some previous studies did not set up a control group, or the control group was not representative enough, such as stomach cancer patients. Inappropriate controls introduced confounding factors in addition to study variables, such as the presence or absence of gastric cancer. Both the lack of a control group and the weak representation of the control group could undermine the validity of their findings. Third, we used PSM analysis in subgroups, which could eliminate or reduce the selection bias or error brought by confounding factors. The BGBD in our study included clinically common subtypes: gallstones, cholecystitis, and gallbladder polyps. Most previous studies used cholelithiasis, cholecystitis, or cholecystectomy as a complete substitute for gallbladder disease and did not conduct PSM analysis.

Our research also had some shortcomings that need to be overcome. As a single-center retrospective study, selection bias and confounding factors could not be avoided, although large sample size and PSM analysis ameliorated part of the bias. In addition, due to lack of information on gender, age, BMI, and comorbidities of control group, we were unable to achieve PSM analysis between case and control groups and only performed PSM analysis in subgroups of case group. We failed to achieve a direct PSM analysis among three subgroups, which is an undisputed shortcoming that might reduce the validity of our results. However, methods supporting direct PSM analysis of the three subgroups were limited at present, and we tried repeatedly, but it was still difficult to achieve. Although the best PSM analysis was not achieved, we believed that results obtained from PSM analysis of the two groups were still convincing. We believe that with the maturity of PSM analysis, multisubgroup direct PSM analysis will eventually be realized. Furthermore, we could not identify whether patients with BGBD and CRC share the same oncogene mutation, because BGBD was not routinely tested for genes. Therefore, although BGBD occurred before CRC in our study, we could not completely rule out effect of causal inversion. To solve this disturbance, Mendelian randomization studies should be needed.

## Conclusion

In conclusion, our study showed benign gallbladder disease was associated with increased risk of colorectal cancer, particularly with right colon cancer. Cholecystectomy was weakly positive with right colon cancer before PSM, but the association disappeared after PSM.

## Data Availability

The raw data supporting the conclusions of this article will be made available by the authors, without undue reservation.
